# Census and vaccination coverage of owned dog populations in four resource-limited rural communities, Mpumalanga province, South Africa

**DOI:** 10.4102/jsava.v88i0.1529

**Published:** 2017-09-22

**Authors:** Anne Conan, Joy A.C. Geerdes, Oluyemisi A. Akerele, Bjorn Reininghaus, Gregory J.G. Simpson, Darryn Knobel

**Affiliations:** 1 Center for Conservation Medicine and Ecosystem Health, Ross University School of Veterinary Medicine, Saint Kitts and Nevis; 2 Department of Veterinary Tropical Diseases, University of Pretoria, South Africa; 3 Game Rangers International in Zambia, Lusaka, Zambia; 4 Gauteng Department of Agriculture and Rural Development, Gauteng, South Africa; 5 Department of Agriculture, Rural Development, Land and Environmental Affairs, Mpumalanga Veterinary Services, South Africa; 6 Department of Production Animal Studies, University of Pretoria, South Africa

## Abstract

Dogs (*Canis familiaris*) are often free-roaming in sub-Saharan African countries. Rabies virus circulates in many of these populations and presents a public health issue. Mass vaccination of dog populations is the recommended method to decrease the number of dog and human rabies cases. We describe and compare four populations of dogs and their vaccination coverage in four different villages (Hluvukani, Athol, Utah and Dixie) in Bushbuckridge Municipality, Mpumalanga province, South Africa. Cross-sectional surveys were conducted in the villages of Athol, Utah and Dixie, while data from a Health and Demographic Surveillance System were used to describe the dog population in Hluvukani village. All households of the villages were visited to obtain information on the number, sex, age and rabies vaccination status of dogs. From May to October 2013, 2969 households were visited in the four villages and 942 owned dogs were reported. The populations were all young and skewed towards males. No differences were observed in the sex and age distributions (puppies 0–3 months excluded) among the villages. Athol had a higher proportion of dog-owning households than Hluvukani and Utah. Vaccination coverages were all above the 20% – 40% threshold required for herd immunity to rabies (38% in Hluvukani, 51% in Athol, 65% in Dixie and 74% in Utah). For the preparation of vaccination campaigns, we recommend the use of the relatively stable dog:human ratio (between 1:12 and 1:16) to estimate the number of dogs per village in Bushbuckridge Municipality.

## Introduction

Dogs (*Canis familiaris*) are raised and kept as domestic animals all over the world. Types of dog management vary and depend on human factors such as cultural or social drivers (Bogel & Hoyte 1990; Knobel et al. 2008). Dog populations are often described by their level of dependency on people and their level of movement restriction (confinement). Based on these criteria, the World Health Organization (WHO) distinguishes four population classes: restricted dogs (these are fully dependent and fully restricted and supervised), family dogs (these are fully dependent and semi-restricted), neighbourhood dogs (these are semi-dependent and semi-restricted) and feral dogs (these are independent and unrestricted) (Bogel & Hoyte 1990). In most sub-Saharan African countries, including South Africa, most of the domestic dogs are free-roaming and owned (Cleaveland 2003; Gsell et al. 2012), and could be considered either family or neighbourhood dogs.

Dogs can be infected by rabies virus, which causes a worldwide zoonotic disease with a high burden in Asia and Africa (Knobel et al. 2005). The disease is deadly for all mammalian species. The main reservoir species of rabies varies across different regions and ecosystems. In South Africa, two biotypes circulate in animal populations: the mongoose biotype occurs in yellow mongooses (*Cynictis penicillata*), while the canid biotype is highly adapted to canine species and can circulate independently in dog populations (Gummow, Roefs & De Klerk 2012; Ngoepe, Sabeta & Nel 2009). In resource-limited communities, where the cycle is maintained by dogs, the main sources of human rabies are dog bites or other contact with saliva of infectious dogs (Cleaveland et al. 2006; Jemberu et al. 2013).

Control of rabies in dog populations leads to a decrease in the incidence of canine-mediated human rabies (WHO 2013). The gold standard method for control of canine-mediated rabies is considered to be mass vaccination of the dog populations (Cleaveland et al. 2006; De Lucca et al. 2013; Lembo et al. 2010; Velasco-Villa et al. 2008). The basic reproductive number (*R*_*0*_) of rabies was estimated at 1.05–1.85; therefore, herd immunity in the dog population would be attained when the proportion of the population that is immune increases from 20% to 40% (Hampson et al. 2009). The level of population immunity in free-roaming dog populations is unstable and difficult to maintain over time because of the high turnover of these populations (frequent exits of vaccinated dogs and frequent entry of non-vaccinated dogs). Thus, the recommendation by WHO is to reach an empirical vaccination coverage of 70% during annual vaccination campaigns, to account for this high turnover (WHO 2013). Simulations based on field studies estimate that annual vaccination coverage of 70% is sufficient to maintain coverage above the predicated threshold for herd immunity during the period between vaccination campaigns, even in high turnover populations (Conan et al. 2015a; Morters et al. 2014). Therefore, knowledge of the size of the dog population is necessary to adequately plan and achieve the target vaccination coverage of 70% of the population.

Conan et al. (2015a) described the dynamics of an owned, free-roaming dog population in a rural village (Hluvukani) in South Africa. The findings indicated that the population was young and male biased, birth and mortality rates were high, and the population was not growing. Reported vaccination coverage in the population remained above 40% over the 2-year study period. However, these results focused on a particular village in the area. Extrapolation of these results to all the resource-limited rural villages of the area would be speculative. Populations of dogs could differ from one village to another, based on, for example, human demographic or social drivers. The objective of this study is to compare dog populations and their rabies vaccination coverage in four neighbouring rural villages (including Hluvukani) in Mpumalanga province.

## Material and methods

**FIGURE 1:**
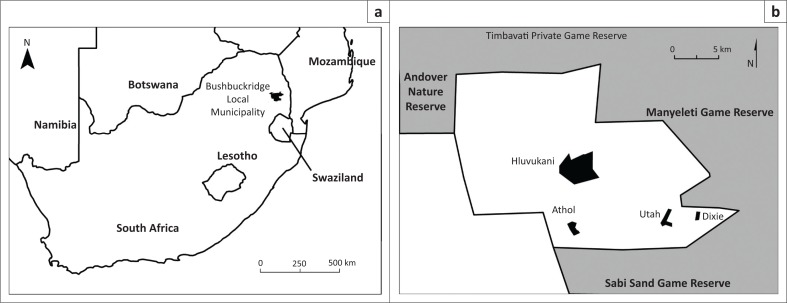
(a) Location of Bushbuckridge Local Municipality in South Africa and (b) location of Hluvukani, Athol, Utah and Dixie villages in the Mnisi Tribal Authority area and proximity of game reserves.

Our study took place in four communities (Hluvukani, Athol, Utah and Dixie) within the eastern part of the Mnisi Tribal Authority’s area, Bushbuckridge Local Municipality, Mpumalanga province, South Africa (Figure 1). Hluvukani community actually encompasses two villages but as the only separation is a road, it was studied as a single entity. The Mnisi Tribal Authority’s area (coordinates: East: 31.35º; South: 24.25º) is adjacent to four game reserves (Andover Nature Reserve [ANR], Timbavati Private Nature Reserve [TPNR], Manyeleti Nature Reserve [MNR] and Sabi Sand Widltuin [SSW]), which are part of the Great Limpopo Transfrontier Conservation Area. The distances from the boundaries of the closest reserve vary: Hluvukani is situated 3 km from MNR, Athol 1.2 km from SSW, Utah 1.2 km and Dixie 0.5 km from MNR.

Rabies vaccination campaigns are performed annually by the Mpumalanga Veterinary Services (MPVS), represented in the study area by the State Veterinary Office Bushbuckridge East-Orpen. While campaigns were historically conducted with varying schedules and intensities, they have followed a designated systematic approach since 2013. All 20 villages of the local State Veterinary area (including the four study villages), as well as 6 further settlements from adjoining State Veterinary areas, covered in a joint approach, are vaccinated over a period of 1 year (February to November). Two to four mobile vaccination teams visit between 100 and 400 households per day. The teams consist of at least two people who proceed door to door. Vaccination certificates are given to the owners for every animal vaccinated. Vaccinated dogs are temporarily marked with crayon. Crayon has a short time of persistence (Conan et al. 2015b). Household gates are also marked by drawing a cross with chalk. The process involves handing out of information leaflets about the disease and recording all dogs seen as well as all dogs vaccinated.

While Hluvukani is the site of the ongoing Health and Demographic Surveillance System of Dogs (HDSS-Dogs), the studies in Athol, Utah and Dixie are the first published dog demographic studies in these villages. Started in 2011, HDSS-Dogs is a longitudinal study set up to monitor the population of dogs in a particular demographic surveillance area (DSA), comprising the approximately 2300 households of Hluvukani village. During regular visits to all households, dog entries (including births) and exits (including deaths) to and from households are recorded. Dogs are identified by subcutaneously implanted electronic microchips (Backhome Biotech, Virbac^®^). If a microchip is not implanted, the dog is identified by its name, sex and household of first observation. A first census of the dogs in Hluvukani was conducted in 2011, as a basis for the HDSS-Dogs. A second census was performed from May to October 2013 during the fifth round of visits of HDSS-Dogs. This latter census was contemporary with the censuses of the other three villages, and is used for the present study.

All households in Athol, Utah and Dixie were visited from July to October 2013. The heads of each household were asked about the dogs they owned. If the head of a household was absent, the household was visited a maximum of two more times. Two different questionnaires were used: one for Hluvukani and another for the other three villages. Common data collected from all households included the number of inhabitants, the number of dogs and sex, age and rabies vaccination status of each dog. Age and rabies vaccination status were owner reported. To calculate the vaccination coverage, the dogs were considered immunised if they were vaccinated within the 3 years prior to the visit (Coyne et al. 2001; Lakshmanan et al. 2006). In Athol, Utah and Dixie, the census team asked to see the rabies vaccination certificate issued by the MPVS. The date of birth was calculated as the midpoint of upper and lower estimations of the time since birth. Questionnaires in Athol, Utah and Dixie included questions about the litters in the past 12 months, the fate of puppies from these litters and causes of deaths in puppies. The two questions about causes of death and fate of puppies were multiple choice questions. Both were asked for all puppies born from the same bitch (non-puppy individual record). Both questionnaires were filled with Open Data Kit (ODK) software on a Samsung Galaxy Tablet. Global Positioning System (GPS) coordinates of all households in Hluvukani were collected with a Garmin GPS^®^, while household coordinates in Athol, Utah and Dixie were collected using the GPS application on the tablet.

Data captured during the study were downloaded from ODK software in comma-separated value files. Analyses were performed with R software (R Core Team 2014). Maps were created with ArcGIS Version 10.2. Geospatial data from the Department of Rural Development and Land Reform were used to estimate village area and distance from the game reserves (Anon 2009).

## Results

From May to October 2013, 2969 households were visited in the four villages. In Hluvukani, of the 2292 households identified by HDSS-Dogs from the round 1 census to the round 4 visit (2011–2013), 1908 (83%) were visited during the round 5 census (2013). In Athol (*n* = 592), Utah (*n* = 390) and Dixie (*n* = 132), the head of the household was absent for 42, 20 and 4 households, respectively. All present heads of households consented to be interviewed. Table 1 describes the human and dog populations in the four villages. The distributions of number of people per household were different between the four villages (Kruskal–Wallis test *p*-value < 0.001). The proportion of dog-owning households (DOHH) was higher in Athol than in Hluvukani and Utah (Chi-square test *p*-value < 0.0001). No differences in dog sex ratio between villages were present (Chi-square test *p*-value = 0.9). Dog age distribution was significantly different in Hluvukani compared to other villages (Kruskal–Wallis test *p*-value < 0.001), but this difference was not present after excluding the puppies aged 0–3 months (*p* = 0.6). Only a few dogs were spayed or neutered (none in Athol, 3 in Dixie, 7 in Utah and 35 in Hluvukani).

**TABLE 1:** Dog and human population description in four resource-limited rural villages at proximity of wildlife conservation areas, Mnisi Tribal Authority, Bushbuckridge Municipality, Mpumalanga province, South Africa (2013).

Dog and human population indicators	Categories	Hluvukani	Athol	Utah	Dixie
Area (km^2^)	-	4.46	0.73	0.74	0.29
Number of interviewed households	-	1908	550	370	128
Percentage of non-respondent households	-	17%	7%	5%	3%
Human population	-	8916*	2889†	1692	484
Human population density (people/km^2^)	-	1999*	3958†	2286	1669
People per household	Minimum	1	1	1	1
	25th percentile	3	3	3	3
	Median	5	5	4	4
	75th percentile	7	7	6	5
	Maximum	23	21	13	13
Owned dog population	-	557	241	107	37
Owned dog population density (dog/km^2^)	-	125	330	145	128
Dog:human ratio	-	1:16*	1:12†	1:16	1:13
Sex of dogs	Male	345 (62%)	149 (62%)	63 (59%)	24 (65%)
	Female	209 (38%)	92 (38%)	44 (41%)	13 (35%)
	Unknown	3	0	0	0
Dog sex ratio (males/females)	-	1.7	1.6	1.4	1.8
Dog age distribution	0–3 months	98 (18%)	5 (2%)	4 (4%)	0
	4–11 months	70 (13%)	39 (18%)	19 (20%)	6 (24%)
	12–23 months	111 (20%)	38 (17%)	12 (13%)	3 (12%)
	24–35 months	99 (18%)	44 (20%)	16 (17%)	4 (16%)
	36 months +	174 (31%)	96 (43%)	43 (46%)	12 (48%)
	Unknown	5	19	13	12
Dogs per household	-	0.29	0.44	0.29	0.29
Number of dog-owning households	-	291	139	53	24
Percentage of total households	-	15%	25%	14%	19%
Dogs per dog-owning household	Minimum	1	1	1	1
	25th percentile	1	1	1	1
	Median	1	1	2	1
	75th percentile	2	2	2	2
	Maximum	18	7	6	3

* , Data of human population are only available for 1814 households (538 dogs);

† , data of human population are only available for 548 households (240 dogs).

Owners in Athol, Utah and Dixie (*n* = 102) reported 192 litters, including 98 in 12 months before the visit, born from owned adult (12+ months) female dogs (*n* = 102; annual litter rate: 0.96) . The median size of litters born in the 12 months preceding the visit was 5 puppies (interquartile range: 3–7; 8 missing data). Of the 398 puppies born from these litters, 161 (40%) died before the visit. The cause of death was reported by the owner as infectious diseases (11; 25% of bitches with report of puppy deaths), stillborn or dead just after birth (7; 16%), accident (5; 11%), another cause (11; 25%) or unknown (10; 23%). Surviving puppies were mostly given away (49; 79% of bitches with litters) or kept in the household (29; 47%).

According to data from Athol, Utah and Dixie, most of the dogs were roaming free with no confinement (279; 72%), whereas fewer dogs were confined to the household’s property either part time (53; 14%) or permanently (53; 14%). None of the dogs were confined in a cage or a kennel.

Overall vaccination coverage at the time of the census was 47% (439/942). Vaccination coverages per village are presented in Table 2. In Athol, Utah and Dixie, three owners reported vaccination but could not recall the vaccine type; their dogs were considered unvaccinated. Only two of the dogs were reported as receiving another injection on top of rabies vaccine during the last 12 months: one combined vaccine (covering canine distemper virus, canine adenovirus 1 and 2, parainfluenza virus and canine parvovirus) and one unknown injection.

**TABLE 2:** Rabies vaccination coverage reported by owner or by Mpumalanga Veterinary Services.

Vaccination indicators	Hluvukani	Athol	Utah	Dixie
Dates of census (2013)	May–October	July–October	July–October	July–October
Number of census dogs	557	241	107	37
Rabies vaccination coverage (proportion of dogs vaccinated at least once in the previous 3 years)	213 (38%)	123 (51%)	79 (74%)	24 (65%)
Vaccinated once or more during a campaign by Mpumalanga Veterinary Services	NA	108 (45%)	71 (66%)	23 (62%)
Presentation of rabies vaccination certificate	NA	56 (23%)	38 (36%)	3 (8%)
Rabies vaccination during the last 12 months	89 (16%)	118 (49%)	69 (64%)	18 (49%)
Dates of Mpumalanga Veterinary Services campaign in 2013	January, July and September	January, March and October	April and June	June
Number of dogs vaccinated in campaign	251	104	97	35
Estimated vaccination coverage achieved by Mpumalanga Veterinary Services	45%	43%	90%	94%

NA, not applicable.

* , All percentages have the total number of dogs in the villages for denominator.

## Ethical considerations

Written informed consent was obtained from a member of each household during the first census of the HDSS-Dogs in Hluvukani (2011) and thereafter orally at each round. Oral informed consent was obtained at Athol, Utah and Dixie. The HDSS-Dogs project, as well as the census of dogs in the three other villages, received the approval of the University of Pretoria’s Animal Ethics Committee (V033-11) and the agreement of the local state veterinarian.

## Discussion

This study describes dog populations in four neighbouring resource-limited rural communities at the boundary of the Great Limpopo Transfrontier Park in South Africa. The dog populations had a rabies vaccination coverage at or higher than the required threshold of herd immunity to rabies (20% – 40%) (Hampson et al. 2009). The vaccination coverage reported by the local MPVS shows a wider range in coverage. These vaccination coverages should be interpreted with caution, as the denominator (total number of dogs in our study) was not collected at the same time period as the vaccination campaign. The number of dogs vaccinated is higher than the owner-reported number. This can be explained by the probable deaths or out-migration of some dogs. In other African countries, a review of rabies vaccination coverage by Jibat, Hogeveen and Mourits (2015) reported coverages of 64% – 80% immediately after vaccination campaigns done with no cost for owners versus below 50% when the vaccination was charged to the owner (Jibat et al. 2015). We presume that the relatively good vaccination coverages in Athol, Utah and Dixie were because of the design (door to door) and the free provision of vaccine (Jibat et al. 2015; Wera, Mourits & Hogeveen 2016).

In 2013, 43 cases of rabies in animals were reported in the Bushbuckridge Municipality (Figure 1), consisting of 32 dogs, 10 cattle and 1 goat (B. Reininghaus [MPVS] pers. comm., 2013). In the same year, 14 rabid dogs were euthanised in the nature reserves bordering the study area, of which 4 were encountered in proximity to the adjacent study area (B. Reininghaus [MPVS] pers. comm., 2013). This shows that rabies virus was circulating in the larger area encompassing the studied villages in 2013. Only two confirmed rabies cases in dogs in the four study villages were reported during 2013, in January and February (B. Reininghaus [MPVS] pers. comm., 2013). There is no proven reason for the subsequent absence of cases in the study area after February for the remainder of 2013. This could be because of herd immunity in the villages or because of the particular spatial pattern of the cases in the municipality, which did not include the study villages during this period. More data, such as spatial distribution of rabies cases in the municipality, are needed to link this absence of cases with the vaccination coverage in the four studied villages.

As could be expected, given the limited resources of the human population, dogs are rarely vaccinated against other diseases (distemper, parvovirus, etc.), for which no cost-free provision is available. Consequently, the endemicity of these diseases is likely to be partly responsible for the high observed mortality of dogs, especially puppies (Conan et al. 2015a; Kolo 2016).

Dog density ranged from 125 dogs/km^2^ to 330 dogs/km^2^. These numbers are high and comparable with density usually observed in peri-urban or urban areas (Kitala et al. 2001; Tenzin et al. 2015). Therefore, the high dog density may be linked with high human density. Dog:human ratio is comparable to or smaller than most of the previous reports in rural areas (Davlin & Vonville 2012). As this ratio is relatively stable among the four villages, this result could be used locally for the estimation of the size of the dog population for vaccination campaigns; however, dog:human ratios should be used with caution. While it is not recommended to use these for vaccination coverage estimates (Sambo et al. 2017), the ratio is a good indicator for vaccine procurement and preparation of vaccine campaigns (Gibson et al. 2016). Thus, we recommend that the local veterinary services use the dog:human ratio (1:15) to estimate the dog population and evaluate the number of vaccine doses to procure to attain 70% vaccination coverage.

The dog populations in the four communities were skewed towards males. This pattern is described in most free-roaming dog populations (Davlin & Vonville 2012). This unbalanced sex ratio is either because of the entry of more males (e.g. a skewed sex ratio at birth [Pal 2001]) or because of the exit of more female dogs from the population (death or movement). As the data were collected in different, adjacent villages, the movement of more female dogs than male ones out of the study area appears unlikely. Owners might kill female dogs more often, if they consider them to be unwanted (Massei et al. 2017). However, the number of human-mediated deaths reported in Hluvukani is low (Conan et al. 2015a; Kolo 2016). Higher specific mortality rates in adult female dogs were observed during a suspected distemper outbreak in Hluvukani village (Conan et al. 2015a), and could be because of natural poorer health condition of female dogs compared to male ones. These two biological hypotheses (skewed sex ratio at birth and higher female mortality) should be further investigated to better understand the sex ratio.

The total population of all four villages was relatively young, with around 60 of the population being younger than 3 years. More puppies were detected in Hluvukani. We suppose this is because of two main factors: firstly, the study time in this village encompassed the birth season (May–July) (Conan et al. 2015a; Kolo 2016) and secondly, the duration of the study in Hluvukani was longer (6 months vs. 4 months in the three other villages). Thus, the study in Hluvukani had a higher probability of recording the presence of puppies, while in the other villages most puppies born during the peak of birth were dead before the study (extremely high mortality in the 0–3 months age group) (Conan et al. 2015a). The age structure in Athol, Utah and Dixie would then be representative of the ‘recruited’ population (dogs that owners wish to keep) as they are not given away or dead. In these three villages, the annual rate of litters per adult female dogs was 0.96. This rate is higher than what was observed in other domestic dog populations, for example, Tanzania or in Zimbabwe, but litter size (five puppies per litter) is comparable (Butler & Bingham 2000; Gsell et al. 2012).

Several limitations in our study have to be noted. Firstly, almost all data were owner reported; consequently, the accuracy of data cannot completely be assessed. As an example, the owner-reported vaccination coverage in Hluvukani is relatively low (38%) compared to the estimation by Conan et al. (2015a) using a longitudinal methodology during the same period (between 50% and 60%). We suspect this difference might be because of owner recall bias during the census in 2013, with owners reporting only recent events. During the launch of the HDSS-Dogs in 2011, recorded dogs were also vaccinated (Conan et al. 2015a). If the owner did not recall this event, the vaccination coverage would be underestimated. Secondly, no investigation of the presence and number of unowned stray dogs was performed, although they are considered to be only a very small proportion of the total population (B. Reininghaus [MPVS] pers. comm., 2016).

## Conclusion

In conclusion, Athol, Utah and Dixie, relatively small rural villages with various human population sizes, had dog populations with demographic characteristics comparable to the dog population of a more urban and relatively highly populated village, studied by HDSS-Dogs (Conan et al. 2015a): young male-biased populations with a high turnover but an adequate rabies vaccination coverage. Each population presented sufficient vaccination coverage to protect the population against a potential rabies outbreak at the time of the study. We can assume that other neighbouring villages present the same type of population. As rabies is still a relevant issue in the area, further measures to support rabies vaccination are needed. While it has been repeatedly shown that mass dog rabies vaccination is the best control measure to eliminate dog-mediated rabies, key demographic factors such as mortality, reproduction and movements need to be further studied and described over a long-term period to understand how to maintain a stable population and enhance the efficiency of dog rabies vaccination campaigns.
